# *Pneumocystis jiroveci* pneumonia in kidney and simultaneous pancreas kidney transplant recipients in the present era of routine post-transplant prophylaxis: risk factors and outcomes

**DOI:** 10.1186/s12882-018-1142-8

**Published:** 2018-11-21

**Authors:** Neetika Garg, Margaret Jorgenson, Jillian Descourouez, Christopher M. Saddler, Sandesh Parajuli, Brad C. Astor, Arjang Djamali, Didier Mandelbrot

**Affiliations:** 1 Division of Nephrology, Department of Medicine, University of Wisconsin School of Medicine and Public Health, 1685 Highland Ave, 4177 Medical Foundation Centennial Building, Madison, WI 53705 USA; 2 Department of Pharmacy, University of Wisconsin Hospital and Clinics, Madison, WI USA; 3 Division of Infectious Diseases, Department of Medicine, University of Wisconsin School of Medicine and Public Health, Madison, WI USA; 4 Department of Population Health Sciences, University of Wisconsin School of Medicine and Public Health, Madison, WI USA; 5 Division of Transplant Surgery, University of Wisconsin School of Medicine and Public Health, Madison, WI USA

**Keywords:** Bactrim, CMV, Kidney transplant, Pneumocystis, Prophylaxis, Sulfamethoxazole-trimethoprim

## Abstract

**Background:**

The goal of this study was to identify predictors for development of *Pneumocystis jirovecii* pneumonia (PJP) in kidney and simultaneous kidney and pancreas transplant recipients in the present era of universal primary prophylaxis.

**Methods:**

We reviewed adult recipients of kidney transplant or simultaneous pancreas and kidney transplant at the University of Wisconsin between January 1, 1994 and December 31, 2016. Patients diagnosed with PJP during this time frame were included. Controls were randomly selected from among those whose post-transplant course was not complicated by PJP, matched on time since transplant through incidence density sampling with a 3:1 ratio.

**Results:**

28 (0.45%) of 6270 recipients developed PJP between 1994 and 2016. Median time since transplant was 4.6 years (interquartile range (IQR): 1.4–9.6 years). Affected recipients were older, had more HLA mismatches, and were more likely to have had BK viremia, CMV viremia and invasive fungal infections than matched controls. CMV viremia remained the only significant risk factor in multivariate analysis, and was a strong predictor (OR 6.27; *p* = 0.002). Ninety percent of the cases with prior CMV viremia had been diagnosed in the year preceding the diagnosis of PJP; among these, median time from diagnosis of CMV to diagnosis of PJP was 3.4 months (IQR: 1.74–11.5 months) and median peak CMV viral load prior to diagnosis of PJP was 3684.5 IU/mL (IQR: 1034–93,300 IU/mL). Additionally, 88.9% of patients with CMV in the preceding year had active infection at time of PJP diagnosis. Patient and graft survival were significantly worse at 2 years in recipients with PJP than our control group (42.4% vs. 88.5, and 37.9% vs. 79.9%; *p* < 0.001).

**Conclusions:**

Despite the low overall incidence of PJP in the era of universal prophylaxis, outcomes are poor. We suggest extending or re-initiating PJP prophylaxis for at least 6 months in the setting of CMV viremia due to the relatively low risk of therapy and potential significant impact on disease prevention.

## Background

*Pneumocystis jirovecii* pneumonia (PJP), previously known as *Pneumocystis carinii* pneumonia, is an opportunistic fungal infection observed in immunocompromised individuals. Prior to institution of routine prophylaxis in kidney transplant recipients, the incidence was reported at 5 to 15% and was the highest in the first 6 months after transplantation [1–6]. Prophylaxis with trimethoprim-sulfamethoxazole (TMP-SMZ) is highly effective and is now routinely administered for the initial 6 to 12 months post-transplantation at most transplant centers in the United States [7]. This has drastically reduced the incidence of PJP during the initial highest risk period immediately following transplantation. However, the epidemiology of PJP in the modern era of universal prophylaxis and risk factors for late onset infection in the renal transplant population have not been fully elucidated. There is no consensus on which high-risk individuals may benefit from prolongation or re-initiation of prophylaxis [8, 9].

At our center, administration of PJP prophylaxis is protocolized for the initial 6 to 12 months immediately following transplant. The primary objectives of this study were: 1) to identify risk factors for PJP that, in turn, could provide guidance regarding when prolongation or re-initiation of prophylaxis may be warranted, and 2) to describe outcomes in kidney transplant and simultaneous pancreas kidney recipients whose post-transplant course has been complicated by PJP.

## Methods

### Study population and design

We reviewed adult recipients of kidney transplant or simultaneous pancreas and kidney transplant at the University of Wisconsin between January 1, 1994 and December 31, 2016. Data were collected from the Wisconsin Allograft Recipient Database (WisARD) and the electronic medical record. Patients were included if they had evidence of PJP disease defined by identification of infiltrates on chest imaging, and documentation of *Pneumocystis jirovecii* by direct immunofluorescence testing or polymerase chain reaction (PCR) technique on sputum or bronchoalveolar lavage fluid samples. Controls were randomly selected from among those whose post-transplant course was not complicated by PJP, matched on time since transplant through incidence density sampling with a 3:1 ratio. This was done due to the broad time range used for the selection of cases, and to be able to account for era effects in use of immunosuppressive regimens and other treatment protocols. This study was approved by the local institutional review board.

### Primary PJP prophylaxis

Since 1983, the first line PJP prophylactic regimen at our center has been TMP-SMZ for a duration of 12 months with dose ranging from 160 mg–800 mg thrice weekly to once daily based on renal function and other variables. In patients with documented allergies to sulfa drugs or in the presence of another contraindication, such as persistent hyperkalemia or leukopenia, a second line agent (atovaquone 1500 mg once daily, dapsone 100 mg once daily or inhalational pentamidine 300 mg once a month) is utilized for 6 months post-transplant.

### Outcomes

Variables collected included demographics (age, sex and race), type of transplant (living donor vs. deceased donor), prior transplantation history, degree of HLA mismatch, induction immunosuppression and delayed graft function. We also recorded data on history of post-transplant complications *prior* to the diagnosis of PJP, or the corresponding time for selected controls, including biopsy proven rejection, BK viremia, CMV viremia, invasive fungal infections, and diarrheal illnesses due to *Clostridium difficile* or norovirus. Since our center uses a threshold of 1000 copies/mL of BK viremia as a cue for immunosuppressive medication adjustment, we used the same threshold to define BK viremia as a risk factor for this study. Additionally, at our center, standardized protocol does not call for CMV PCR testing unless there is clinical concern for CMV viremia. Therefore, any detectable viremia (> 250 IU/mL or equivalent copies/mL) was used to define CMV viremia, in order to capture all occurrences. Invasive fungal infections included aspergillosis, cryptococcosis, histoplasmosis, blastomycosis and coccidioidomycosis [10].

### Statistical analysis

Differences in the demographic and clinical characteristics of transplant recipients who developed or did not develop PJP were examined through use of t-tests or chi-square tests, as appropriate. Logistic regression along with the vce (cluster *clustvar*) command available in Stata was used to test statistical significance of various comparisons, while taking into account the clustering due to incidence density sampling. Multivariate logistic regression analysis was used to identify risk factors for development of PJP after adjusting for the potential confounding risk factors identified based on a significant univariate relationship defined by a *p*-value of less than 0.05.

To compare clinical outcomes in transplant recipients with PJP to those without PJP, the data was censored at the time of last available follow up with a functioning graft. Kaplan-Meier analyses were done to analyze graft and patient survival. All statistical analyses were conducted using Stata MP 13.0 (StataCorp, College Station, TX).

## Results

### Diagnosis of PJP

**Fig. 1:**
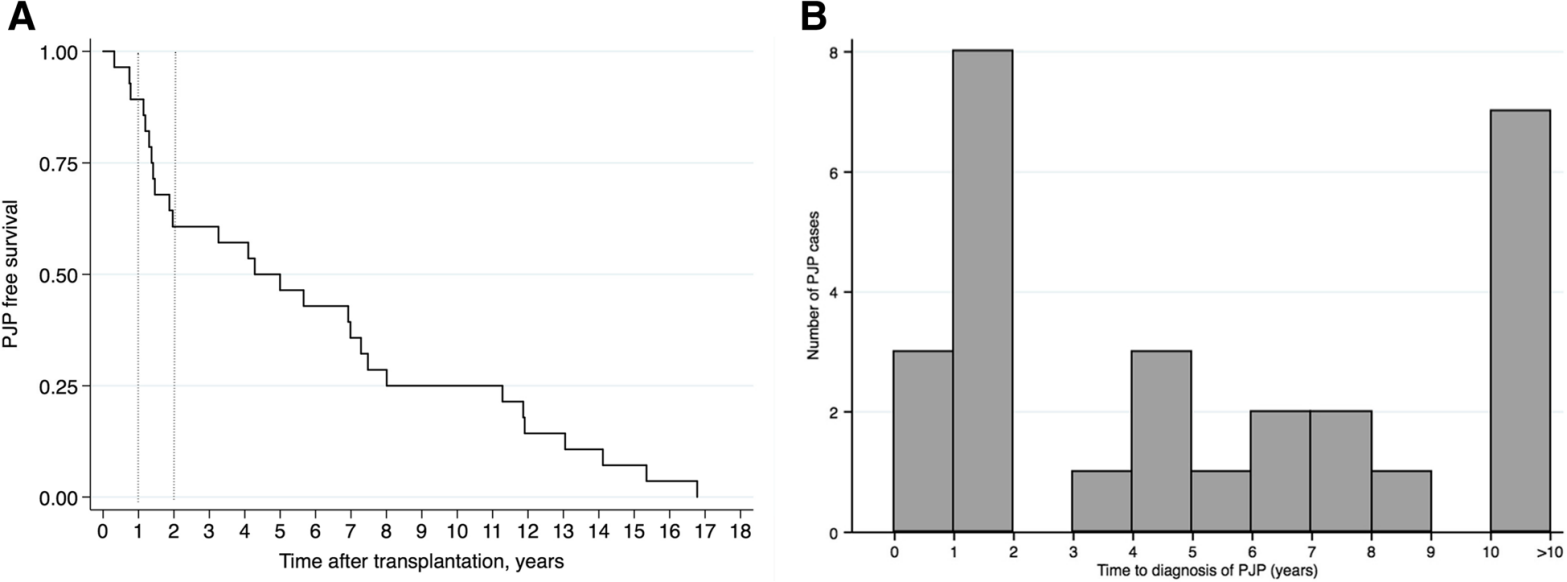
**a** Time to development of PJP after transplantation. The year two delineated between the dotted lines represents the year with the highest incidence of PJP infections. **b** Histogram for time to diagnosis of PJP

A total of 6270 kidney-alone and simultaneous pancreas and kidney transplants were performed during the study period. Of these, 28 (0.45%) recipients, including 4 recipients of simultaneous kidney and pancreas transplants, were diagnosed with PJP, yielding an incidence rate of 0.065 per 100 person-years. Median time from transplant to diagnosis of PJP was 4.6 years (interquartile range (IQR) 1.4 to 9.6 years) (Fig. 1a). Notably, 10.7% (*n* = 3) cases of PJP were diagnosed within the first year (Fig. 1b). Each of these three patients was off prophylaxis at the time of developing PJP. Incidence rate of PJP in the first year after transplantation was 0.051 per 100 person-years. The second year after transplantation had the highest proportion of PJP, with almost a third of the total cases diagnosed during this time (28.6%, *n* = 8) and incidence rate of 0.152 per 100 person-years. Incidence rate of PJP anytime after the second year post-transplantation was 0.053 per 100 person-years. A quarter of the cases (25.0%, *n* = 7) were diagnosed more than 10 years after transplant.

### Baseline characteristics and predictors of PJP

Mean age of the total study population (*n* = 112) was 47.7 years. Approximately 60% were men and 86.6% were non-Hispanic whites. More than half received an allograft from a deceased donor (56.3%) and 80% were recipients of a primary transplant.

The differences in baseline characteristics between the PJP and the matched controls are summarized in Table 1. Overall the groups were well matched from the standpoint of sex, race, donor type, prior transplants, induction immunosuppression and delayed graft function after transplant (Table 1). On univariate analysis, transplant recipients with PJP infection were older (mean age 52.5 years vs. 46.2 years; *p* = 0.03), and had higher number of HLA mismatches (65.4% vs. 35.7% had > 3 mismatches, with 3 being the median for the entire group; *p* = 0.005). The incidences of prior BK viremia (21.4% vs. 6.0%; *p* = 0.01) and CMV viremia (35.7% vs. 6.0%; *p* < 0.001) were higher in the PJP group. Patients in the PJP group were also more likely to have had invasive fungal infections (7.1% vs. 1.2%; *p* = 0.01). There was no significant difference in incidence of preceding diarrheal illness due to *Clostridium difficile* or norovirus between the two groups (10.7% vs. 2.4%, *p* = 0.11). Additionally, there was no difference in the rate of prior biopsy proven allograft rejection between the two groups (39.3% vs. 29.8%, *p* = 0.41).

**Table 1:** Comparison of various clinical characteristics between the PJP and the non-PJP groups

	Non-PJP group (*n* = 84)	PJP group (*n* = 28)	*P*-value
Age at time of transplant (years)	46.2	52.5	0.03*
Female (%)	44.0	28.6	0.17
Non-white (%)	9.5	25.0	0.07
Living donor transplants (%)	47.6	32.1	0.14
History of prior transplantation (%)	21.4	15.4	0.41
Induction immunosuppression			0.22
- None	13.1	7.7	
- Basiliximab	36.9	65.4	
- Thymoglobulin	21.4	7.7	
- Alemtuzumab	13.1	19.2	
- Other	15.5	0	
HLA mismatch	2.7	3.9	0.005*
- HLA mismatch > 3 (%), with 3 being the median for the entire group	35.7	65.4	
Delayed graft function (%)	11.9	14.3	0.76
BK viremia (%)	6.0	21.4	0.01*
CMV viremia (%)	6.0	35.7	< 0.001*
Biopsy proven rejection (%)	29.8	39.3	0.41
Invasive fungal infections (%)	1.2	7.1	0.01*
Clostridium difficile or norvirus diarrheal illness (%)	2.4	10.7	0.11
- Norovirus (%)	0	3.6	
- Clostridium difficile (%)	2.4	7.1	

*P*-values less than 0.05 are highlighted with an*

Of the variables associated with PJP in univariate analysis i.e. age, HLA mismatch, prior BK viremia, CMV viremia and invasive fungal infections, only CMV viremia remained significantly associated after multivariate adjustment (OR 6.27, *p* = 0.002) (Table 2).

**Table 2:** Predictors of PJP occurrence using logistic regression

Variables	Odds ratio	95% Confidence interval	*P*-value
Age (per 10 years increase)	1.35	0.91–2.01	0.14
HLA mismatch > 3	2.41	0.95–6.12	0.06
BK viremia	1.53	0.23–10.20	0.66
CMV viremia	6.27	1.94–20.23	0.002*
Invasive fungal infections	2.10	0.92–4.83	0.08

Model included variables identified as significantly associated with PJP on univariate analysis. *P*-values less than 0.05 are highlighted with an*

Importantly, 90% of the CMV viremic patients who went on to develop PJP had CMV viremia in the year preceding the diagnosis of PJP (Table 3). Among these (*n* = 9), median time from diagnosis of CMV viremia to diagnosis of PJP was 3.4 months (IQR: 1.74–11.5 months), and median peak CMV viral load prior to diagnosis of PJP was 3684.5 IU/mL (IQR: 1034–93,300 IU/mL). 88.9% (*n* = 8) were on active treatment for CMV viremia at time of PJP diagnosis: four were receiving valganciclovir treatment and had detectable viremia, three were receiving valganciclovir maintenance therapy and were not viremic, and one had low grade viremia that was being monitored without antiviral therapy. 11.1% (*n* = 1) had cleared viremia and completed valganciclovir therapy.

**Table 3:** Detailed clinical characteristics of patients with CMV viremia who were subsequently diagnosed with PJP

	Time from transplant to PJP diagnosis (years)	Time from CMV viremia to PJP diagnosis (months)	Peak CMV viral load in the year prior to PJP diagnosis (IU/mL)	Viremia present at time of PJP diagnosis	Valganciclovir therapy at time of PJP diagnosis	Ganciclovir-resistant CMV
Patients with PJP who had CMV viremia in the year preceding diagnosis of PJP						
1	11.9	0.9	826	No	Yes	No
2	7.3	3.0	6105	Yes	Yes	No
3	7.5	1.7	1264	No	Yes	No
4	1.1	0.9	1150	Yes	Yes	Yes
5	1.3	11.8	93,300	Yes	Yes	No
6	1.4	11.5	387	Yes	No	No
7	11.9	5.6	184,559	No	Yes	No
8	1.9	11.8	139,535	No	No	No
9	0.8	3.4	1034	Yes	Yes	No
Patients with PJP who had history of CMV viremia more than one year prior to diagnosis of PJP						
1	16.8	92.1	NA	NA	NA	No

### Outcomes after PJP

**Fig. 2:**
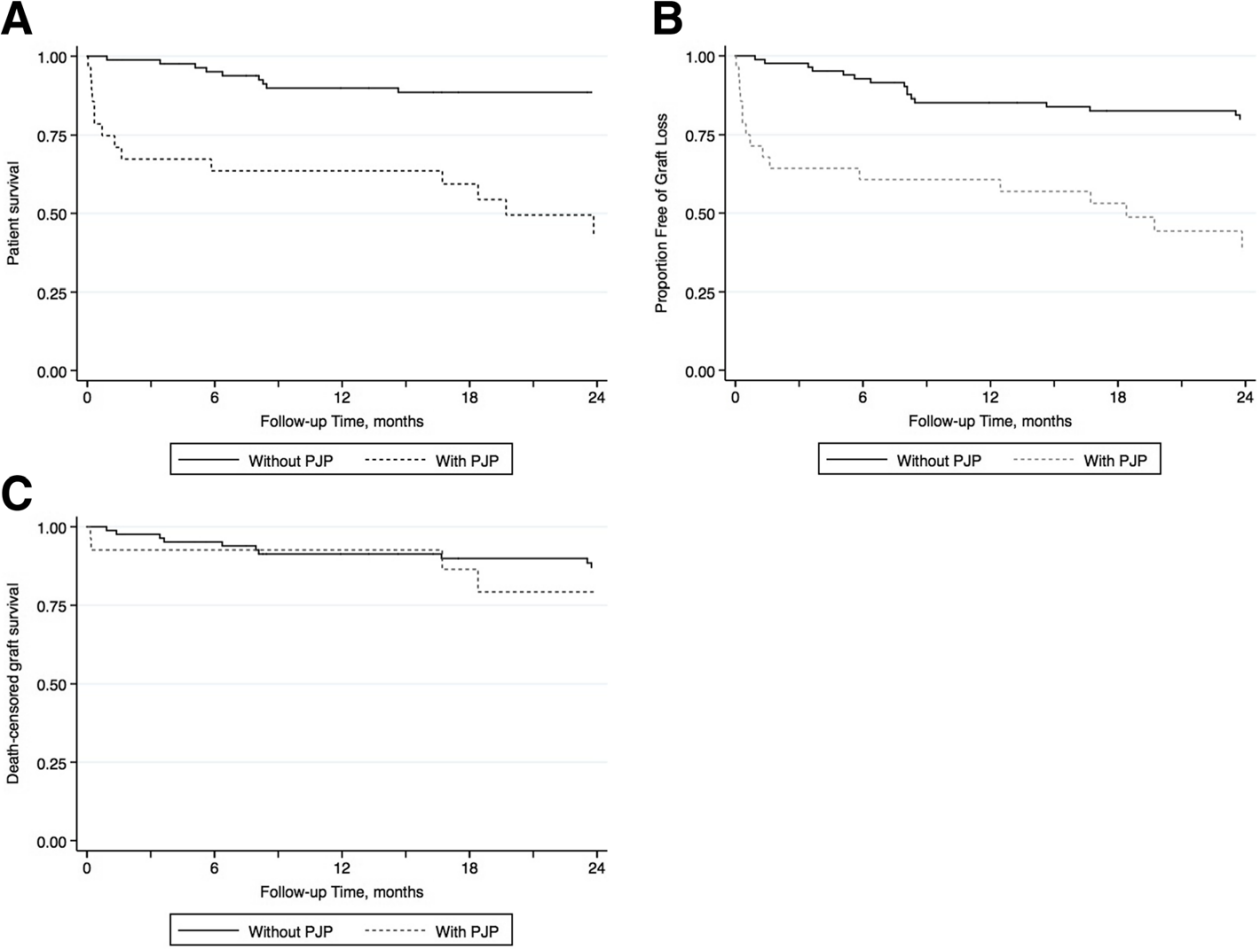
**a** Kaplan-Meier curve for patient survival in transplant recipients with and without PJP (*p* < 0.001) ‘Follow-up time’ represents time since diagnosis of PJP in transplant recipients with PJP, and corresponding time point in controls selected using incidence density sampling. **b** Kaplan-Meier curve for graft survival in transplant recipients with and without PJP (*p* < 0.001). ‘Follow-up time’ represents time since diagnosis of PJP in transplant recipients with PJP, and corresponding time point in controls selected using incidence density sampling. **c** Kaplan-Meier curve for death-censored graft survival in transplant recipients with and without PJP (*p* = 0.316). ‘Follow-up time’ represents time since diagnosis of PJP in transplant recipients with PJP, and corresponding time point in controls selected using incidence density sampling

Patient survival was significantly worse at 3 months (62.3% vs. 95.2%; *p* < 0.001; Fig. 2a) and at 2 years (42.4% vs 88.5%; *p* < 0.001) in those with PJP than in their counterparts without PJP. Similarly, graft survival also was worse at 2 years in those with PJP (37.9% vs. 79.9%; *p* < 0.001, Fig. 2b). No significant difference in death-censored graft survival was observed (79.2% vs. 87%, *p* = 0.316, Fig. 2c). Lastly, no patient had a second episode of PJP.

## Discussion

The present study from a high-volume transplant center spanning the course of over two decades provides several insights into the epidemiology of PJP in kidney and simultaneous kidney and pancreas transplant recipients in the present era of routine post-transplant prophylaxis. First, the overall incidence of PJP in the setting of protocolized universal prophylaxis in the initial 6–12 months is low, at less than 1 %, compared to the historically reported statistic from pre-prophylaxis era of 5 to 15% [1–6]. A recent retrospective analysis of all solid organ transplant recipients from the Swiss Transplant Cohort Study reported an incidence of PJP of 1.4%; the incidence was higher at 3.2% in the 13.3% of the recipients who did not receive primary prophylaxis vs. 1.2% in those who received primary prophylaxis [11]. In our study, only three cases of PJP were reported in the first year following transplantation. In each of these, prophylaxis with TMP-SMZ had been stopped prematurely due to medication adverse effects. Additionally, consistent with recent studies, the immediate post-prophylaxis period, i.e. the second year post-transplant, has replaced the first year post-transplant as the highest risk period for developing this opportunistic infection with almost a third of the cases diagnosed during this time [8, 11–13]. Interestingly, this trend is similar to what is observed in the CMV literature, where routine prophylaxis has postponed the highest risk period [11, 14]. It should be highlighted that this change does not reflect just a shift in the period of peak PJP risk, and that the incidence of PJP (overall as well as in the second year) is extremely low compared to the pre-prophylaxis era. Also, PJP can occur very late after transplantation, with a quarter diagnosed a decade after transplant in our study.

Secondly, CMV viremia is a clinically strong and statistically significant predictor of subsequent PJP infection [8, 9, 11–13, 15–18]. Our study was not designed to identify which patients with CMV viremia, a common infection after transplantation, subsequently develop PJP, a rare complication [3]. Nonetheless, it is noteworthy that over 90% of the transplant recipients with PJP who had history of prior CMV viremia had been diagnosed with CMV in the year preceding diagnosis of PJP, and almost all of these were still viremic and/or receiving treatment at the time of admission for PJP. Median time from diagnosis of CMV viremia to diagnosis of PJP was 3.4 months, which argues against simple detection of stress-induced viremia in the setting of impending PJP syndrome. Previous literature echoes our findings; Iriart and colleagues previously reported a 52% incidence of CMV viremia in the year preceding PJP in a study of all solid organ transplants, including kidney, heart and liver [8]. Lee and colleagues reported a median time between CMV viremia and PJP infection of almost 2 months [9]. These results from our and prior studies provide an opportunity to identify this high-risk cohort; we suggest consideration of extending or re-initiating prophylaxis for at least 6 months in recipients whose course is complicated by CMV viremia. Lastly, while history of BK viremia and invasive fungal infections were more common in transplant recipients who developed PJP, the significance of relationship between these and PJP was lost on multivariate analysis. However, extension or re-initiation of prophylaxis should also be considered in recipients with CMV viremia and BK or fungal co-infections.

The association between rejection and risk of subsequent PJP remains unclear. Contrary to what might be expected and findings from some other investigations [13, 18], rejection did not prove to be significantly associated with subsequent diagnosis of PJP. This may be a result of confounding by the protocolized use of 3 months of PJP prophylaxis in the setting of rejection treatment at our center, and is consistent with some recent studies [8, 9]. Similarly, older age was not a predictor of PJP in our study. Findings from previous studies are divided, with the Iriart and Neofytos studies describing age greater than 65 years as a risk factor, and the Lee study demonstrating no significant difference [8, 9, 11].

In addition to rejection and older age, several other variables frequently used as proxies for the degree of immunosuppression, including T-cell depleting induction and BK viremia, were not significant risk factors for subsequent diagnosis of PJP. These results suggest that the increased risk of PJP in recipients with CMV viremia may be a consequence of the substantial immunomodulatory effects of the virus, rather than a reflection of the overall level of immunosuppression alone [19].

Finally, despite a reduction in overall incidence in the modern era, patient as well as graft outcomes continue to be poor among recipients whose course is complicated by PJP [20]. Over half of our patients with PJP died within 2 years. This underscores the importance of identifying patients at high risk for PJP who are likely to benefit from highly effective PJP prophylaxis with TMP-SMZ or alternate agents.

This study has all the limitations of being a small series from a single center. However, data on all our transplant patients is collected prospectively, we analyzed matched controls and our database is one of the few in the country that would be large enough to provide this series. Secondly, individual maintenance immunosuppressive regimens were not analyzed. However, immunosuppressive regimens have been fairly consistent throughout the study time period with a triple drug regimen of a calcineurin-inhibitor, antimetabolite and corticosteroid being our standard of care. Thirdly, preceding lymphopenia has been previously shown to be a risk factor for PJP [8]. Corresponding data was missing for many of the PJP cases in our study and therefore could not included in our analysis. Lastly, the number of PJP cases may appear small, however, PJP is now a rare complication of kidney transplantation, and to the best of our knowledge, this is the largest study of kidney transplant recipients from the present era of routine TMP-SMZ prophylaxis.

## Conclusions

In conclusion, in our review of over 6000 transplant recipients, routine PJP prophylaxis has drastically reduced the incidence of PJP to less than 1 %. The peak incidence time period has also been shifted from the initial 6 months to the second year after transplant. Patient survival in affected patients remains dismal, with 2-year mortality rate approaching 60% with treatment. CMV has consistently been shown to be a strong predictor of subsequent PJP and because of this, we suggest extension or re-initiation of PJP prophylaxis for at least 6 months in recipients with CMV viremia. Future studies are needed to elucidate the impact of this intervention on rates of PJP in those with CMV viremia, as well as the feasibility of this approach in this population from tolerability from a myelosuppression standpoint.

## Abbreviations

IQR: Interquartile range
OR: Odds Ratio
PCR: Polymerase chain reaction
PJP: *Pneumocystis jirovecii* pneumonia
TMP-SMZ: Trimethoprim-Sulfamethoxazole
